# Emerging Role of Hippo-YAP (Yes-Associated Protein)/TAZ (Transcriptional Coactivator with PDZ-Binding Motif) Pathway Dysregulation in Renal Cell Carcinoma Progression

**DOI:** 10.3390/cancers16152758

**Published:** 2024-08-03

**Authors:** Varsha Mondal, Paul J. Higgins, Rohan Samarakoon

**Affiliations:** Department of Regenerative and Cancer Cell Biology, Albany Medical College, 47 New Scotland Avenue, Albany, NY 12208-3479, USA; mondalv@amc.edu

**Keywords:** YAP/TAZ, kidney cancer, clear cell RCC, MST, LATS1/2, core hippo pathway, growth factors, TEAD, tumorigenesis

## Abstract

**Simple Summary:**

The mechanisms of renal cell carcinoma (RCC) progression are not well understood. There is a need to develop effective therapies against metastatic disease and to reduce high disease prevalence among minority and male populations. Here, we detail limited but compelling evidence for the role of the Hippo-YAP (Yes-associated protein)/TAZ (transcriptional coactivator with PDZ-binding motif) pathway in RCC progression. YAP/TAZ activation is evident in human RCC. Inhibition of YAP/TAZ in mice with kidney cancer mitigates neoplastic behavior, highlighting the therapeutic benefit of YAP/TAZ targeting in RCC. This review also summarizes, for the first time, upstream controls and oncogenic consequences associated with Hippo-YAP/TAZ dysregulation in progressive RCC. We describe critical literature gaps and future studies necessary to position the Hippo pathway among well-established drivers of kidney cancer progression.

**Abstract:**

Although Hippo-YAP/TAZ pathway involvement has been extensively studied in the development of certain cancers, the involvement of this cascade in kidney cancer progression is not well-established and, therefore, will be the focus of this review. Renal cell carcinoma (RCC), the most prevalent kidney tumor subtype, has a poor prognosis and a high mortality rate. Core Hippo signaling inactivation (e.g., LATS kinases) leads to the nuclear translocation of YAP/TAZ where they bind to co-transcriptional factors such as TEAD promoting transcription of genes which initiates various fibrotic and neoplastic diseases. Loss of expression of LATS1/2 kinase and activation of YAP/TAZ correlates with poor survival in RCC patients. Renal-specific ablation of LATS1 in mice leads to the spontaneous development of several subtypes of RCC in a YAP/TAZ-dependent manner. Genetic and pharmacological inactivation of YAP/TAZ reverses the oncogenic potential in LATS1-deficient mice, highlighting the therapeutic benefit of network targeting in RCC. Here, we explore the unique upstream controls and downstream consequences of the Hippo-YAP/TAZ pathway deregulation in renal cancer. This review critically evaluates the current literature on the role of the Hippo pathway in RCC progression and highlights the recent scientific evidence designating YAP/TAZ as novel therapeutic targets against kidney cancer.

## 1. Introduction

### Renal Cell Carcinoma Prevalence, Histological Subtypes, and Clinical Challenges

Renal cell carcinoma is the most common form of urologic cancer accounting for 2–3% of all malignant diseases. RCC is the seventh most prevalent cancer in men and the ninth in women [1]. Although RCC is relatively rare compared to other cancers, the steadily growing incidence and higher prevalence among males and minority populations requires additional investigation to design therapy targets [2]. Molecular characterization identified several subtypes of RCC based on histopathological evidence. Clear cell RCC (ccRCC) is the prevalent RCC histological subtype (70–80% cases) and entails the highest mortality. ccRCC derives from the proximal convoluted tubules and presents as a solid yellowish lesion with different degrees of internal necrosis, cystic degeneration, and tumorous calcifications [3]. This tumor subtype is composed of distinctly clear cells due to their lipid and glycogen-rich cytoplasmic content and sparse organelles [4]. The presence of hypervascularized and heterogenous lesions due to necrosis and hemorrhages makes this cancer more invasive and lethal. Approximately 10–15% of RCCs are papillary tumors (pRCC). The spindle-like organization of cells and internal hemorrhages, cystic alterations, and larger lesions are distinctive features of this subtype. Chromophobe RCC (chRCC), which originates from the renal distal tubules, has a 5% prevalence, and contains a pale reticular cytoplasm with prominent cell borders. chRCC has a low somatic mutation rate compared to ccRCC and is less aggressive. Sarcomatoid renal cell carcinoma is a highly dedifferentiated tumor with spindle-like morphology. This aggressive tumor type has high metastatic potential with poor disease outcomes in patients. ccRCC progression is associated with a Von Hippel–Lindau (VHL) tumor suppressor loss-of-function mutation [5,6], leading to the activation of HIF1 and HIF2, which drive carcinogenesis [7]. Several oncogenic pathways such as HIF1α, VEGF, and MET, as well as immune checkpoint failure, are associated with RCC progression and poor prognosis [8]. Metastatic renal carcinoma patients have a 5-year survival rate (less than 10%) despite advancements in targeted treatments including anti-angiogenic drugs and immune checkpoint inhibitors [9]. The mechanisms of disease progression are not fully understood, highlighting the need for the development of efficient therapeutic options to increase disease-free survival.

## 2. Hippo Pathway Components and Their Role in Physiology

**Figure 1:**
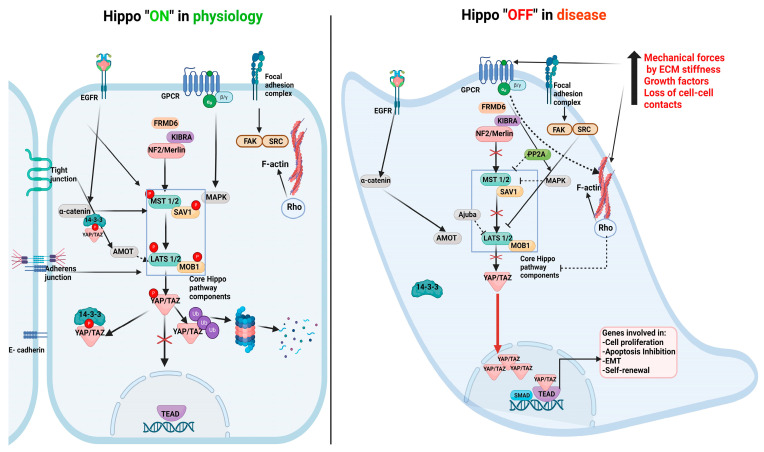
Schematic representation of Hippo “ON” signaling in physiological conditions and Hippo “OFF” signaling in disease states. Upstream signals emanating from tight and adherens junctions and kinases such as NF2/Merlin and KIBRA stimulate phosphorylation of core Hippo kinases, MST, and LATS1/2. The counterparts of the KIBRA/Merlin/FRMD6 complex exist in drosophila while there is evidence for KIBRA and Merlin interaction in mammals. Subsequent LATS-dependent phosphorylation of the downstream effectors, YAP/TAZ, can result in their cytoplasmic sequestration or proteasomal degradation, thereby restricting growth and maintaining organ size. In disease states, cell–cell junctions are disrupted which can lead to acquisition of mesenchymal phenotype and ECM modifications. Increased mechanical forces, upregulation of various growth factors, and cytokine and cytoskeletal remodeling initiated signaling events that could lead to inactivation of the core Hippo pathway cascade, resulting in YAP/TAZ nuclear translocation and accumulation. YAP/TAZ requires co-transcription activators such as TEADs or SMADs to initiate transcriptional responses of target genes pertinent to cell proliferation, dedifferentiation, self-renewal and apoptosis inhibition. Red X: implies inactivation.

As an evolutionarily conserved signaling network, the Hippo pathway is a major determinant of tissue growth and organ size during embryonic development by regulating cell proliferation and apoptosis and influencing tissue regeneration [9,10,11]. This pathway comprises the core elements as well as nuclear effectors. Core Hippo pathway components include MST1/2, LATS 1/2, SAV1, and MOB1. This signaling cascade is activated by high cell density leading to phosphorylation of MST1/2 and SAV1, which form a complex with and activate LATS1/2 by phosphorylation. pLATS1/2 in turn induces phosphorylation of YAP and TAZ (nuclear effectors of the Hippo pathway) on serine residues, resulting in their cytoplasmic retention by binding to the 14-3-3 protein or protein degradation. Since YAP and TAZ do not have DNA-binding domains, they orchestrate target gene transcription by interacting with co-activators such as TEAD. Hippo signaling can be regulated by cell shape and polarity, cell–matrix adhesion, cell–cell interactions, cytoskeletal alterations, mechanical forces, the tissue microenvironment, and various cytokines and growth factors, which can influence YAP/TAZ nuclear translocation and subsequent transcriptional outcomes. YAP/TAZ nuclear accumulation and binding to TEAD leads to the transcriptional activation of genes that mediate cell proliferation, EMT, and apoptosis (Figure 1).

Inhibition of YAP/TAZ nuclear translocation prevents cell proliferation, thereby restricting organ growth and maintaining organ size [10]. The Hippo pathway can also be modulated by various growth factors and their receptors including EGFR, TGF-beta, Wnt, and their downstream signaling effectors Rho-GTPases, Merlin, KIBRA, RASSFs, and Ajuba [11,12,13,14]. Cell–cell contact mediated by adherens junctions or tight junctions leads to YAP/TAZ cytoplasmic retention, mediated by 14-3-3 binding, α catenin, ZO-2 proteins, and E-cadherin, which leads to the inhibition of cell growth [11]. Cell polarity and cytoskeletal changes further contribute to Hippo pathway activation [12] (Figure 1). Precise controls of the Hippo pathway in the progression of certain cancers such as RCC remain elusive.

## 3. Hippo Pathway Dysregulation in Renal Disease Progression

The ability of YAP/TAZ/TEAD complexes to regulate cell growth, death, and epithelial plasticity contributes to several cancers and the development of tissue fibrosis [13,14,15,16]. Hippo pathway perturbations are associated with tumorigenesis due to hyperactivation of YAP/TAZ signaling [13]. Loss of cell density or polarity, and increased mechanical forces in the tumor microenvironment, disable the activity of core Hippo components with subsequent YAP/TAZ activation [17]. Renal epithelial-specific Mst1/2 gene ablation in mice results in YAP nuclear accumulation and spontaneous renal fibrosis [18]. Nuclear accumulation of YAP is evident in polycystic kidney disease patients, and kidney epithelial cell-specific YAP overexpression in mice stimulates cell proliferation and the formation of renal cysts [19]. YAP/TAZ regulation by mechano-signaling induces TGF β-induced SMAD2/3 signaling in fibroblasts and the development of renal fibrosis [20]. YAP/TAZ can also reprogram tumor cells to assume stemness and promote EMT, tumor formation and metastasis [21,22]. These findings implicate YAP/TAZ as potential oncogenes [23,24]. Although Hippo pathway involvement is described in tumor progression in some organs [25], the role of this pathway in kidney cancer is not well understood. Therefore, this review highlights specific upstream control and downstream pathogenic roles of Hippo-YAP/TAZ dysregulation in RCC progression using evidence from animal models, cell culture studies, and human diseases.

### Direct Evidence for Core Hippo Pathway Inactivation in Human Kidney Cancer and Mouse Models of RCC Progression

Hippo network inactivation is evident in several histopathological subtypes of RCC. The Cancer Genome Atlas (TCGA) database analysis revealed that core Hippo pathway inactivation is associated with worse outcomes in human RCC patients [26]. Dysregulation of Hippo signaling activates several oncogenic pathways [26]. Renal tubular expression of LATS 1/2 is dramatically downregulated during the progression of RCC in human specimens. Such findings suggest, therefore, that certain Hippo pathway intermediates could serve as renal tumor suppressors under normal circumstances since their loss of expression may predispose them to RCC progression [26]. The LATS1 promoter is also highly hypermethylated in ccRCC tumors, leading to decreased mRNA and protein levels of LATS1. Repression of LATS1 as well as YAP1 overexpression, in fact, correlates with poor outcomes in ccRCC patients [27]. Genomic landscape assessments by targeted panel sequencing of patient tumor samples also identified frequent alterations of the Hippo pathway in sarcomatoid RCC (sRCC).

A recent elegant study confirmed that the inactivation of Lats1/2 in the kidney epithelium in rodents is sufficient to promote metastatic RCC progression [28]. Inducible genetic ablation of Lats 1/2 in the proximal renal tubules in adult mice (generated by a crossing of tamoxifen-inducible proximal tubule-specific Cre line -Slc34a1CreERT2 with Lats1/2 floxed mice) resulted in the formation of a high-grade sarcomatoid RCC phenotype within 4 months with metastasis to the lung [28]. Sarcomatoid renal cell carcinoma is an aggressive form of RCC [29]. Tubular-specific genetic dual depletion of YAP/TAZ in Lats1/2-deficient mice, conversely, mitigated tumor formation, directly demonstrating YAP/TAZ involvement in renal tumorigenesis downstream of Lats 1/2 deficiency. Transcriptome analysis of proximal tubule-derived Lats1/2 mutant tumors in mice revealed derivation of different histological subtypes including clear cell RCC and chromophobe RCC, suggesting that Lats1/2 pathway repression can give rise to several RCC subtypes. Collectively, these studies demonstrated the significance of the core Hippo kinase dysregulation and the oncogenic effects of YAP/TAZ activation in RCC progression.

## 4. Downstream Consequences of YAP Hyperactivation in RCC Progression

Immunohistochemistry (IHC) assessments of YAP1 and Lats1 protein levels in 54 human tumor sections of ccRCC and normal renal tissues revealed prominent YAP1 nuclear immunoreactivity in the proximal tubules of ccRCC tumors compared to the normal kidney [30]. Lats1 immunoreactivity, more evident in the cytoplasm, negatively correlated with ccRCC tumor size. Multivariate Cox analysis on the tumors demonstrated that increased YAP1 expression and decreased Lats1 immunoreactivity are associated with worse prognoses in RCC patients and decreased survival [30]. YAP overexpression and nuclear accumulation, indeed, are evident in various types of human RCC. ccRCC cells also have a higher nuclear expression of YAP compared to normal kidney proximal tubules. YAP protein levels in ccRCC cell lines, 786-O and ACHN, are significantly higher compared to human normal kidney epithelial cells. Two recent studies indicate that YAP depletion in 786-O RCC cells significantly attenuated proliferation, migration, and anchorage-independent growth as well as xenograft growth in vivo compared to controls [30,31], which suggests that YAP plays an important role in RCC progression. YAP downregulation resulted in p53 activation and cell cycle inhibition, and YAP depletion of ccRCC cells (786-O) also mitigated subcutaneous xenograft growth [32]. YAP silencing in ccRCC cells similarly led to proliferative defects, G1 cell cycle arrest, and increased apoptosis [33], reducing cell migration while increasing apoptosis in the Caki 2 and 786-O RCC cell lines.

**Figure 2:**
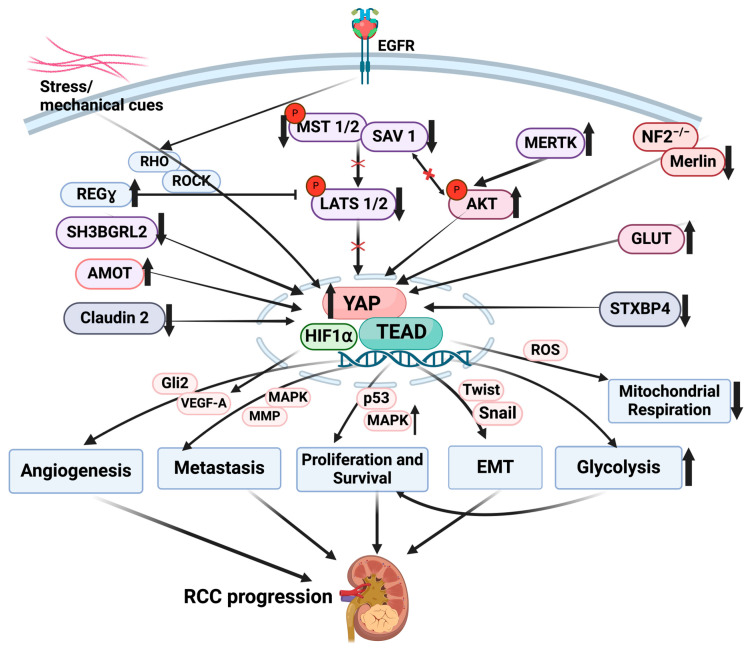
Upstream controls and downstream consequences of Hippo-YAP pathway activation in RCC progression. Mechanical cues and cellular shear stress induce YAP nuclear accumulation and transcription. YAP-mediated upregulation of Snail and Twist transcription factors results in EMT in RCC cells. Growth factors like EGFR upregulation during cancer progression activate Rho GTPases which regulate YAP signaling. YAP is also activated by MERTK through Akt mobilization. NF2/Merlin upregulates YAP during RCC progression, which can orchestrate glycolysis activation and mitochondrial respiratory defects. Amot interacts with YAP, maintains its nuclear accumulation and promotes YAP-mediated RCC proliferation. During hypoxic conditions, YAP upregulation and association with HIF1α induces VEGF-A, thereby increasing angiogenesis and RCC progression. ↑ implies upregulation; ↓ implies downregulation; Red X implies inactivation.

Angiomotin (*Amot*) protein interacts with YAP maintaining nuclear YAP accumulation, leading to RCC proliferation [34]. Depletion of Amot inhibited cell proliferation in 786-O cells, while overexpression of YAP in the Amot-silenced ccRCC cells restored cancer cell proliferation. Amot knockdown also reduced the transcription of YAP/TAZ target genes CTGF and Cyr61 in 786-O cells. Therefore, Amot supports YAP-dependent cancer cell proliferation [34] (Figure 2).

Increased mechanical forces are linked to cancer progression and shear stress, increasing YAP’s nuclear accumulation in 786-O cells. YAP knockdown attenuates stress-induced downregulation of E-cadherin, suggesting that YAP plays a role in EMT. YAP depletion also mitigates stress-induced upregulation of Snail and Twist (two major EMT-promoting transcription factors), correlating with reduced cellular plasticity [35]. YAP-depleted RCC cells subjected to shear stress are also more prone to cell death. Shear stress-induced YAP nuclear localization is mediated by Rho/Rock-dependent mechanisms. YAP1 therefore serves as a mechanosensor that transduces mechanical stimuli to intracellular signals to promote survival, EMT and tumor progression in RCC cells.

ccRCC is a highly vascularized tumor, and gene set enrichment analysis revealed potential involvement of YAP in tumor angiogenesis in RCC. Additionally, YAP knockdown decreased VEGFA protein levels in ccRCC cells, suggesting that YAP promotes VEGF expression. Silencing of YAP in ccRCC cells (786-O and ACHN) cocultured with HUVEC cells decreased HUVEC recruitment and the formation of tube-like structures in Matrigel. YAP knockdown not only inhibited tumor formation but also neo-vascularization, confirming the participation of YAP in the tumor angiogenesis of RCC cells in vivo. Gene set enrichment analysis (GSEA) indicated a relationship between YAP and Gli2, which is also highly expressed during angiogenesis. Coculture studies showed that the knockdown of Gli2 also abrogated HUVEC recruitment, implicating the role of YAP in promoting tumor angiogenesis by enhancing Gli2/VEGFA expression in RCC cells [36].

Ectopic YAP induction in certain RCC cell lines, however, can reduce proliferation and promote apoptosis in vitro and decrease xenograft growth in vivo [31]. TEAD4 transcription factor, highly induced in TCGA data sets, correlates with poor disease outcomes in RCC patients [31]. TEAD4 depletion, indeed, attenuated proliferation and migration in renal cancer cell lines and xenograft growth in vivo, highlighting an important role of TEAD4 in RCC progression [31]. TEAD4 can interact with NF-κB/p65, and it appears that excess YAP can disrupt NF-κB/p65 association with TEAD4, which could lead to tumor-suppressive effects. Such context-specific effects of YAP in RCC progression (e.g., too much or too little YAP causes defective tumor growth) require further clarification as downstream outcomes of YAP may be dependent on TEAD-binding partners (e.g., p65) and the competition in protein–protein interactions which can influence pathogenic outcomes.

## 5. Downstream Consequences of TAZ Dysregulation in RCC Progression

TAZ expression is similarly elevated in RCC tissues compared to normal tissues [37], correlating with poor patient survival, high Fuhrman grade, invasion, and metastasis, suggesting involvement of TAZ in human RCC. Increased nuclear levels of TAZ are also correlated with high clinical-stage renal cancer tissues. TAZ depletion in RCC cell lines inhibited cell proliferation, cell cycle progression, and migration, promoting apoptosis in vitro, and inhibited tumor growth in mice [38]. Sustained expression of TAZ in renal epithelial cells is sufficient to promote epithelial dedifferentiation (plasticity), marked by a loss of epithelial junction protein, E-cadherin, gain of the mesenchymal marker, vimentin, and expression of various profibrotic and inflammatory factors, including connective tissue growth factor (CTGF) and ECM molecules [39].

Expression of the tumor suppressor gene, RB1CC1 (RB1-inducible coiled-coil protein 1), is lower in RCC tissues compared to normal tissues [37], and increased RB1CC1 levels are linked to better survival in RCC patients. RB1CC1 downregulates the proto-oncogene PYK2, which is highly upregulated in RCC patients.

**Figure 3:**
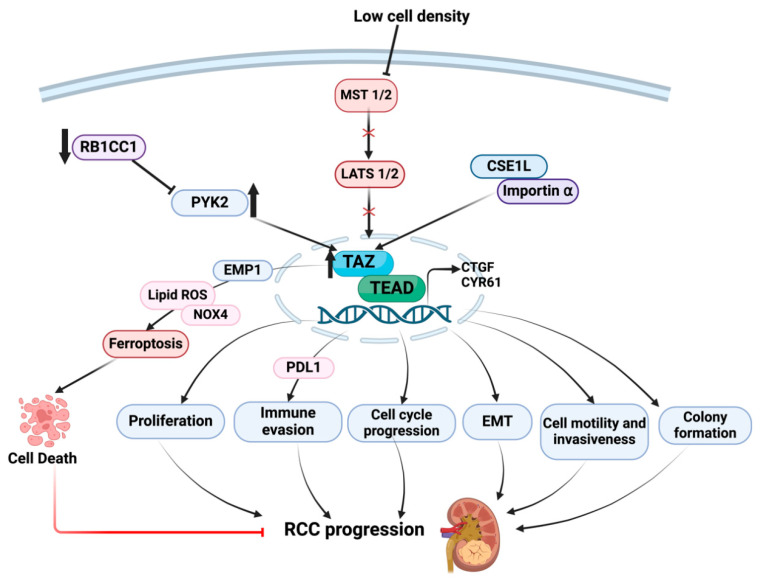
Upstream control of Hippo-TAZ pathway activation leading to renal tumorigenic phenotypes (e.g., proliferation, cell motility, invasion and EMT). Downregulation of the tumor suppressor gene RB1CC1 activates the proto-oncogene PYK2 which, in turn, promotes TAZ protein stability and stimulates immune evasion mediated by CsE1L gene overexpression. In contrast, low cell density promotes TAZ nuclear levels upregulating, thereby EMP1, lipid ROS, and NOX4-mediated ferroptosis of RCC cell lines. TAZ overexpression in RCC cells causes EMT, cell proliferation, and colony formation, leading to RCC progression. ↑ implies upregulation; ↓ implies downregulation; Red X implies inactivation.

PYK2 promotes RCC progression via increased TAZ protein stability and PYK2 stimulates the transcription of the immune PD-L1 gene, thereby promoting immune evasion [37]. TAZ silencing, moreover, reduces PD-L1 transcription and tumor size [37]. Collectively, these findings suggest that TAZ regulates RCC aggressiveness via controlling tumor immunity (Figure 3).

Chromosomal segregation 1-like (CSE1L) overexpression is associated with a poor prognosis in human cancers. Nuclear accumulation of TAZ is a major determinant of its oncogenic activity. CSE1L mediates the nuclear import of TAZ by forming a complex with importin α and CSE1L and utilizes TAZ as a downstream effector in RCC progression as silencing of CSE1L decreases TAZ expression. TI-4 (a TAZ inhibitor) prevents interaction between TAZ and importin α, thereby inhibiting TAZ nuclear localization. Depletion of TAZ also attenuates CSE1L-mediated colony formation, motility, and invasiveness in human cancer cells [40], indicating functional cooperation between CSE1L in TAZ in tumor progression (Figure 3).

Ferroptosis is a form of cell death that is influenced by cell density, and low population density promotes ferroptosis. TAZ nuclear entry is also dependent on cell confluence, and low-density-mediated TAZ activity appears to influence RCC cell ferroptosis. Low density promotes TAZ nuclear levels, which upregulate epithelial membrane protein 1 (EMP1) and NADPH oxidase, NOX4. Reactive oxygen species (ROS) generated by NOX4 appear to promote ferroptosis in RCC lines, suggesting that TAZ impacts cell death in a density-dependent manner. Different mechanisms by which TAZ exerts anti-tumorigenic properties (e.g., ferroptosis) or oncogenic behavior (e.g., proliferation, EMT, invasion) during RCC are also not understood. Additional studies are necessary to determine context-specific upstream signals and TAZ-associated molecules or binding partners that dictate differential outcomes in the context of kidney cancer.

## 6. Upstream Controls on YAP/TAZ Activation in RCC Progression

### 6.1. Neurofibromin 2 (NF2)/Merlin

RCC development is frequently associated with NF2 pathway repression [41]. Core Hippo pathway dysregulation is linked to the generation of both low- and high-grade renal neoplasms in different RCC subtypes [42,43]. Loss-of-function mutations in NF2 resulted in the inactivation of the Hippo core kinase cascade, nuclear translocation of YAP oncoprotein and transcription of genes involved in proliferation and apoptosis inhibition in drosophila [44]. Loss of NF2 induces the formation of neurofibromatosis II and benign tumors, and truncating mutations in Lats2 and NF2 are evident in non-VHL-mutated ccRCC. Whole genome sequencing of primary RCC tumors and cell lines revealed that about 33% of VHL wild-type clear cell RCC had mutations in the NF2 tumor suppressor gene [45]. Genetic studies implicated Merlin as an upstream regulator of the Hippo signaling pathway [44,46]. Deletion of the Nf2 gene in proximal convoluted tubules in mice resulted in the downregulation of Merlin, which prevents MST phosphorylation, thereby activating YAP/TAZ and promoting RCC progression [47]. YAP/TAZ depletion in Nf2-deficient mice, furthermore, represses tumor formation. YAP/TAZ deletion also reduced glycolysis in RCC tumors and increased mitochondrial ROS development, which resulted in oxidative stress-induced cell death. TCGA analysis of primary kidney tumor transcriptomes also revealed a direct correlation of YAP overexpression with glycolysis and an inverse relationship between YAP and mitochondrial oxidative phosphorylation [48]. These data suggest that NF2/merlin is an upstream regulator of YAP during RCC progression, and YAP accumulation due to Nf2 loss promotes tumor growth via increased glycolysis and defective mitochondrial respiration. Whole genome and transcriptome sequencing of mucinous tubular and spindle cell RCC carcinomas (a rare cancer type) also revealed correlations between loss of Hippo pathway tumor suppressor genes such as NF2 and SAV1 and YAP1 nuclear accumulation during cancer progression [49].

### 6.2. Salvador Homolog 1 (SAV-1)

Salvador, a core Hippo kinase pathway component, can regulate organ size by restricting the nuclear translocation of YAP/TAZ, thereby controlling cell proliferation [50]. In high-grade ccRCCs, SAV1 is downregulated by the loss of copy number [51]. Sav1 gene silencing leads to YAP activation and enhanced cell growth, colony formation, and apoptosis inhibition in 786-O cells [51]. Knockdown of SAV1 in HK-2 and RPTEC (primary renal proximal tubular epithelial cells) promoted cell proliferation [51]. SAV1 transduction in ccRCC cells, conversely, resulted in reduced transcriptional activity of YAP1 and TEAD3 [51]. These studies suggest that the downregulation of Sav1 in high-grade ccRCC tumors could promote disease progression via YAP/TEAD3 signaling.

### 6.3. Hypoxia

Although the relationship between VHL and YAP1 in RCC progression is not clear, YAP1 could be associated with HIF-1α (a major downstream target of VHL) in cancer cells under hypoxic conditions to induce VEGF-A transcription [52]. In normoxia, prolyl hydrolase 2 is associated with specific proline residues of YAP, facilitating its interaction with VHL and leading to YAP ubiquitination and degradation [52]. Under hypoxic conditions, the interaction between YAP and VHL is disrupted, leading to YAP upregulation and YAP association with HIF-1α to mediate RCC progression [52], likely via VEGF-A induction and increased angiogenesis.

### 6.4. Proteasome Activator (REGγ)

Upregulation of the proteasome activator REGγ is associated with poor prognosis of RCC [53]. Depletion of REGγ suppressed RCC growth in vitro and in vivo. REGγ knockdown also activated the core Hippo pathway components LATS1 and MST1 by stabilizing casein kinase1ε (CK1ε) and repressing RCC progression [53], suggesting that REGγ upregulation promotes RCC by disabling the Hippo pathway. Additional studies are necessary to understand the mechanistic basis downstream of oncogenic signaling by REGγ.

### 6.5. MER Proto-Oncogene Tyrosine Kinase (MERTK)

MERTK appears to regulate Akt kinase that in turn interacts with SAV1 (via the proline-rich domain), restricting Akt movement to the plasma membrane and inhibiting its activation [54]. Mutations in SAV1 lead to Akt hyperactivation and uncontrolled cancer growth [54]. MERTK disrupts Akt-SAV1 interaction by direct Akt phosphorylation, leading to Akt-mediated kidney tumor cell survival and oncogenesis. MERTK upregulation is linked to poor prognosis in RCC patients [54].

### 6.6. Syntaxin-Binding Protein 4 (STXBP4)

STXBP4 functions as a tumor suppressor in ccRCC. WW domain-containing proteins bind to PY motifs in YAP [55]. STXBP4 is a WW domain-containing protein and represses YAP activity by sequestering it in the cytoplasm by the assembly of PY motif proteins and α-catenin [55]. STXBP4 depletion attenuates YAP phosphorylation, thereby activating YAP-mediated RCC cell proliferation [55], suggesting that STXBP4 is a negative regulator of YAP during RCC tumorigenesis. STXBP4 loss of expression, indeed, correlates with renal carcinoma progression.

### 6.7. SH3-Binding Glutamate-Rich Protein-like 2 (SH3BGRL2)

Bioinformatics and tissue microarray analysis revealed that SH3BGRL2 is a potential regulator of RCC formation and metastasis [56]. SH3BGRL2 plays a critical role as an inhibitor of cell proliferation, migration, and EMT by inactivating Hippo/TEAD1 signaling. SH3BGRL2 downregulation is evident in ccRCC metastatic tumors and correlates with poor prognosis. SH3BGRL2 depletion promotes proliferation and metastasis in 786-O ccRCC cells and in mice by activating the YAP/TAZ-TEAD pathway [56]. SH3BGRL2 loss also promotes Snail and Twist1 activity [56]. SH3BGRL2, therefore, can function as a RCC tumor suppressor by preventing YAP/TAZ-TEAD1 pathway activation.

### 6.8. Claudin-2

Exploration of patient databases revealed that claudin-2 downregulation correlated with an aggressive RCC phenotype and poor survival [57,58]. Claudin-2 loss in ex vivo cultured mouse kidneys led to EMT and increased invasive potential. Overexpression of claudin-2 in RCC-derived tumor cells repressed tumor formation/growth in mouse xenografts. Mechanistically, claudin-2 associates with and phosphorylates YAP at Ser127 residue, thereby preventing its nuclear accumulation [57]. Loss of Claudin-2, therefore, promotes RCC malignancy through YAP activation [57] (see Figure 2).

## 7. Conclusions, Therapeutic Relevance, and Future Directions

There is limited but compelling evidence to establish a role for Hippo pathway dysregulation in renal cancer progression. Loss of expression of core Hippo pathway components and overexpression or nuclear accumulation of YAP and TAZ are evident in various RCC subtypes which correlate with tumor grade and poor patient survival [15,17,23,27]. Proximal tubular-specific Lats1/2 depletion results in spontaneous RCC development in rodents, revealing a crucial role for core Hippo pathway inactivation in disease onset [28]. Depletion of YAP/TAZ in mice with renal tubular-specific Lats1/2 ablation, conversely, attenuated kidney cancer development, revealing the oncogenic role of YAP/TAZ activation in RCC progression [28]. A YAP/TAZ inhibitor, Verteporfin, moreover, mitigated RCC progression in LAT1/2 double-knockout animals.

Since YAP and TAZ orchestrate several downstream effects, including EMT, migration, proliferation, tumor growth, angiogenesis, and immune evasion (see Figure 2 and Figure 3), YAP/TAZ targeting could be a novel and viable approach to slow RCC progression. However, the feasibility of YAP/TAZ inhibition as a potential add-on therapy (for the existing first line of therapy) for progressive human RCC has not yet been tested. Since YAP and TAZ are activated in various forms of renal cancer (e.g., ccRCC), additional studies are necessary to further investigate disease-specific or context-specific (e.g., onset or late-stage) effects of Hippo nuclear effector activation to oncogenesis. Furthermore, the relationship between Hippo pathway alterations and major drivers of ccRCC development (such as VHL, SETD2, and BAP2 mutations as well as HIF1 and HIF2α activation) is not currently clear. Whether Hippo pathway dysregulation correlates with VHL, SETD2, or BAP2 mutations during RCC, and/or YAP/TAZ activation in such settings increases the risk of disease development, requires additional studies. What is also not clear is whether YAP and TAZ can exert different renal oncogenic functions, as evident in other types of cancers [59].

Several renal upstream regulators that mediate core Hippo pathway inactivation and YAP/TAZ cellular distribution have been identified by recent studies. In this regard, NF-2 regulates RCC progression by impacting the core Hippo pathway as NF-2 expression loss in mice prevents Merlin-mediated phosphorylation of MST kinase, which leads to YAP-dependent oncogenic metabolic alterations [44,46]. Therefore, restoration of NF-2 or Merlin could be a potential approach to slow RCC progression. Although mechanisms are not fully understood, REGγ and MERTK also appear to impact core Hippo pathway components during kidney cancer progression [53,54]. STXBP4 and Claudin-2, moreover, can affect YAP phosphorylation and cytoplasmic retention during progressive RCC [57]. Upstream regulators that prevent renal YAP and TAZ nuclear accumulation or induce YAP/TAZ degradation can potentially mitigate subsequent oncogenic responses. Identification of upstream molecules as well as downstream targets that regulate YAP/TAZ hyperactivation provide additional and indirect avenues to suppress Hippo-mediated RCC progression.

## Abbreviations

Amot	Angiomotin
Ajuba	protein encoded by JUB gene
ccRCC	Clear cell Renal cell Carcinoma,
CTGF	CCN2 or Connective Tissue Growth Factor
EGFR	Epidermal Growth Factor Receptor
ECM	Extracellular Matrix
EMT	Epithelial-Mesenchymal Transition
FMR1	Fragile X Messenger Ribonucleoprotein 1
GPCR	G-protein Coupled Receptors
KIBRA	Kidney and Brain Expressed Protein
HIF1α	Hypoxia Inducible Factor α
HUVEC	Human Umbilical Vein Endothelial Cells
HK2	Human Kidney 2
Lats1/2	Large tumor suppressor kinase 1
MET	Mesenchymal Epithelial Transition
MOB1	Mps One Binder 1
MERTK	MER proto-oncogene tyrosine kinase
MST 1/2	Serine Threonine like kinases
mTOR	Mammalian Target of Rapamycin
PYK2	Protein Tyrosine Kinase 2
RASSF	Ras association domain family
RB1CC1	RB1-inducible coiled-coil protein 1
RHO	Ras Homologous
ROS	Reactive Oxygen Species
SAV1	Salvador Homolog 1
STXBP4	Syntaxin-Binding Protein 4
SH3BGRL2	SH3-Binding Glutamate-Rich Protein Like 2
TAZ	Transcriptional coactivator with PDZ-binding motif
TCGA	The Cancer Genome Atlas
TEAD	Transcriptional Enhancer Associated Domain
TGF-β	Transforming Growth Factor β
VEGF	Vascular Endothelial Growth Factor
VHL	Von Hippel–Lindau
Wnt	Wingless/Integrated
YAP	Yes-associated protein
ZO-2	Zonula Occludens 2
